# Mechanisms Matter: A Demonstration of the Importance of Mechanistic Analysis for Evidence Synthesis Using Two Case Studies

**DOI:** 10.1111/jep.70712

**Published:** 2026-11-04

**Authors:** Sahanika Ratnayake

**Affiliations:** ^1^ Department of Philosophy University of Manchester Manchester England

## Abstract

**Rationale:**

Despite a growing recognition of the importance of mechanisms, driven largely by the EBM+ movement, there appears to be little understanding of how to include mechanistic analysis in evidence synthesis.

**Aims and Objectives:**

To demonstrate the importance of mechanistic analysis for the appraisal of study quality and evidence review.

**Method:**

Two case studies – masking to prevent the spread of respiratory illnesses and talking therapy for treatment resistant depression – are used to illustrate the issues arising from appraising outcome research without considering mechanisms.

**Results:**

Mechanistic analysis is important for: designing high‐quality RCTs, appraising their quality (with Risk of Bias tools, GRADE analyses and more broadly) as well as other areas of evidence synthesis.

## 1 Introduction

There is a growing interest in drawing on mechanistic evidence in evidence synthesis. This push has been largely driven by proponents of EBM+ who argue that consideration of mechanisms can enrich the methodology of Evidence Based Medicine (EBM) [1], which tends to exclude or downgrade mechanistic evidence [2]. Outcome evidence – usually in the form of randomised controlled trials (RCTs) and aggregations thereof in the form of systematic reviews and meta‐analyses — are usually the only evidence considered when establishing the efficacy and effectiveness of interventions. However, even the most stalwart champions of the orthodox EBM approach acknowledge that mechanistic analysis can be useful for making judgements about external validity and implementation. The importance of mechanisms is acknowledged in other areas. For example, the SPIRIT checklist for RCT protocols includes a section directing authors to explain the rationale for the study including details of how the intervention might work [3]. At the level of evidence review and synthesis, the Cochrane handbook advises considering mechanisms of action, particularly for complex interventions [4].

Despite the increasing interest in mechanisms, in published studies and evidence reviews, they tend to feature inconsistently or in minimal detail. This may be because it is currently unclear where exactly mechanistic analysis would be useful. Using two case studies – masking to prevent the spread of respiratory illnesses and talking therapy for treatment resistant depression (TRD) – detailed examples of how mechanistic analysis is important for a number of areas will be demonstrated, including: designing high‐quality RCTs, appraising their quality (with Risk of Bias (ROB) tools, GRADE analyses and more broadly) and other areas of evidence synthesis.

### 1.1 A Clarification on Mechanisms

There is a vast literature, both philosophical and scientific, on mechanisms and how they are to be understood. For instance, they can be understood in terms of a complex arrangement of entities and activities with reference to the system of which they are a part [5] or in terms of a spatio‐temporal process [6]. Mechanisms also function at multiple levels, for instance a complex mechanism describing how a mental health disorder arises may make reference to neurotransmitters at a molecular level as well as behavioural risk factors such as social isolation. Additionally, it is important to distinguish between evidence for a particular mechanistic account and the account itself, in order to avoid mechanistic accounts degenerating into unevidenced ‘just‐so’ stories [7, 8].

Though these nuances are significant and important, the following analysis is not a contribution to the theoretical and conceptual literature on mechanisms – the aim is to offer a concrete, practical demonstration of the importance of mechanistic analysis for a previously underexplored area. Though the importance of mechanistic evidence for establishing causal claims with regard to the efficacy and effectiveness of interventions and for resolving problems of external validity is well‐known [8], there is scant discussion of the role of mechanistic analysis in appraising study quality particularly in the context of evidence synthesis.

Below I will use a minimal definition of mechanism and mechanistic analysis, drawing on the preceding nuances where necessary (this approach bears most resemblance to a recent metaphysically minimalist philosophical account of mechanisms as relevant to scientific practice [9]). By mechanisms and mechanistic analysis then, I refer to a theoretical account encompassing: how an intervention might work; how the condition being targeted comes to arise, is transmitted or maintained; factors that may counteract or attenuate the intervention. This theoretical account may include multiple levels of explanation (molecular, physiological, behavioural) and require evaluation of the evidence for the proposed mechanistic account, issues which will be discussed below.

### 1.2 Background

The following is a sub‐project of a wider UKRI‐funded project developing a novel interdisciplinary method of evidence synthesis involving mechanistic analysis. The research team's expertise was diverse, ranging from medicine, public health, philosophy of science and medicine, sociology of science and medicine, science and technology studies, environmental science, psychology, mental health and philosophy of psychiatry.

The primary case study for the project, on which the novel method was developed and tested, was the efficacy of masking to prevent the spread of respiratory illnesses such as COVID‐19 as described in the protocol paper [10]. Other case studies – in law [11], environmental science, drug development and mental health [12] reflecting the team's expertise – were used to explore the wider usefulness of mechanistic analysis and limitations in the current methods of study appraisal and evidence synthesis. For the primary case study extensive analysis of the individual studies and existing systematic reviews were conducted [13, 14], which was not possible for the others. The following is a brief, illustrative analysis based on the author's expertise in the conceptual and methodological issues in the evidence base for psychological therapy, using the additional case of psychological therapy for addressing treatment resistant depression.

In order to demonstrate the importance of mechanistic analysis at both the level of individual studies and evidence synthesis, a key trial for both case studies was considered alongside the Cochrane review for that intervention. The key trials were chosen based on their deep engagement with mechanisms in the study design. The Cochrane review was analysed in light of lessons from the key trial i.e. how does the Cochrane review treat the key trial and its mechanistic insights? The analysis aims to highlight the blind spots and compromises to rigour that followed from insufficient mechanistic analysis.

For any study there will be a multitude of relevant mechanisms. As it was not possible to consider all mechanisms, the focus for each trial was a cluster of mechanisms, particularly related to optimising study design. The criteria for this mechanism cluster was that they: 1) guided basic study design choices i.e. they were impossible to avoid when designing and conducting the trial; 2) plausibly made a difference in the results i.e. they are not superfluous to the outcome of the trial; 3) are not typically considered in generic checklists, ROB tools or GRADE analyses.

Note that just as a collection of RCTs can produce heterogenous results leaving the overall conclusion unclear, evidence for mechanistic accounts is sometimes similarly ambiguous. While some mechanisms have been evidenced to a high degree of certainty, others remain contested by experts in the field. Below, the mechanisms believed to be least contested based on current research were utilised. Despite potential disagreement with regard to specific mechanism accounts, I will show that it is difficult to make even basic design choices for RCTs – for example, how long to run the trial for or when the intervention should be used – without tacitly subscribing to some mechanistic account. At the moment, the mechanistic accounts guiding these design choices are opaque. At the very least, the analysis below will demonstrate the necessity of being transparent about these tacit mechanistic considerations.

### 1.3 Case Study 1: Masking in Reducing Transmission of Respiratory Illnesses

*Key study: MacIntyre et al. 2013 A Randomised Clinical Trial of Three Options for N95 Respirators and Medical Masks in Health Workers*.

The paper reports a 3 arm cluster RCT conducted in Beijing during the 2009–2010 flu season comparing three masking policies in healthcare settings: 1) continuous use of surgical masks while on shift 2) continuous use of N95 respirators while on shift 3) targeted use of N95 respirators while doing high‐risk procedures. In contrast to the widely used surgical masks, N95 respirators are made to specific engineering standards and have a superior filtration capacity and seal around the face [15, 16, 17]. The first two arms correspond to common hospital policies to wear surgical masks continuously while on shift and to use N‐95 respirators in a targeted manner during high‐risk ‘aerosol‐generating procedures’ such as intubation where there is assumed to be a high chance of the patient producing pathogenic particles that persist and remain viable in the air. Further details and results of the trial are summarised in Table 1 below.

**Table 1:** Masking trials.

	Study design	Primary outcomes and results	Discussion of transmission mechanisms	Mechanism informed analysis
1. Loeb et al. 2009 [18]	Two arm noninferiority trial: surgical masks versus N‐95 respirators. *N* = 446 (nurses in 8 tertiary care Ontario hospitals from a range of departments) Masks worn when within 1 m of a patient with febrile respiratory illness^a^ and during high‐risk procedures. Fit testing conducted. Independent observation to confirm adherence but incomplete (did not enter patient rooms, no auditing in emergency departments)	1. Laboratory confirmed Influenza: 2.8% in the surgical mask group versus 1.8% in the N95 group (absolute risk difference: −0.93%; 95% CI: −3.82% to 1.97%; *p* = 0.75). 2. Fourfold rise in serum antibodies: 8.0% in the surgical mask group versus 11.9% in the respirator group (absolute risk difference: 3.89%; 95% CI, −1.82% to 9.59%; *p* = 0.18). **Conclusion: Non‐inferiority of surgical masks to respirators.**	Discussion of all three mechanisms. Droplet described as main mechanism (p.1870). Better fit of respirators considered unimportant (ibid.)	Targeted use of all masks (only when within 1 m of patient) may have been insufficient to capture differences between masks given possibility of aerosol transmission. Note that in light of behavioural mechanisms, targeted use relies on individuals accurately identifying risk.
2. MacIntyre et al. 2009 [19]	Three arm prospective cluster randomised trial: control versus surgical masks versusP2 respirators. *N* = 286 adults in Sydney from 143 households with an infected child. Adults wore masks when in the same room as sick child (index case). No fit‐testing. Independent observation to confirm adherence with interviews to note barriers to adherence.	1. ILI in any household member: 22.3% in the surgical mask group versus 15.2% in the respirator group versus 16.0% in the control group. 2. Laboratory confirmed respiratory viral infection in any household member: Viral pathogens were isolated 6.4% of the surgical mask group, 8.7% in the respirator group versus 3.0% in the control group. **Conclusion: ITT analysis by households and by participants showed no significant difference between the groups.**	Discussion of all three mechanisms (pp. 235, 239). Stated that the main mechanism is unclear and may vary across illnesses. Hand to face contact is also noted as a potential mechanism (pp. 239–240).	Continuous use when in proximity to index case may have improved ability to register differences in arms, but actual rate of adherence low (By day 5, adherence rates were only 31% and 25%, surgical masks vs respirators respectively) with some parents sleeping maskless in the same room as index case. Issues with adherence prevent any conclusion about relative efficacy.
3. MacIntyre et al. 2011 [20]	Three arm cluster randomised trial: surgical masks versus N95 fit‐tested respirators vs non fit‐tested respirators versus convenience, non‐randomised control group. *N* = 1441 Healthcare workers in 15 Beijing tertiary care hospitals from emergency and respiratory wards. Masks used continuously while on shift. Instructions for proper use provided but fit‐testing only in second arm. Independent observation to confirm adherence with interviews to note barriers to adherence. Adherence also recorded on daily diary cards.	1. CRI: 6.7% in the surgical mask group versus 4.6% in the fit‐tested N‐95 versus 3.3% in the non fit‐ tested N‐95 versus 9.2% in the no masks group. 2. ILI: 0.6% in the surgical mask group versus 0.2% in the fit‐tested N‐95 versus 0.4% in the non fit‐ tested N‐95 versus 1.3% in the no masks group. 3. Laboratory‐confirmed viral respiratory infection: 2.6% in the surgical mask group versus 1.7% in the fit‐tested N‐95 versus 1% in the non fit‐ tested N‐95 versus 3.1% in the no masks group. 4. Laboratory‐confirmed influenza A or B: 1.0% in the surgical mask group versus 0.7% in the fit‐tested N‐95 versus 0% in the non fit‐ tested N‐95 versus 1.3% in the no masks group. **Conclusion: The non‐fit tested N‐95 group showed significant differences for CRI in an ITT analysis but not for any other outcomes. In a comparison with the control group the odds ratio for the non‐fit tested N‐95 respirators was significant for CRI and Lab confirmed viral infections.**	Yes, discussion of all three mechanisms (pp. 171–172). States that the main mechanism is unclear and may vary across illnesses and in varying hospital settings. Filtration efficiency of different types of masks also mentioned (p. 171).	The study design accommodates a wide range of potential transmission mechanisms. Though there are differences between arms, they were not consistently significant. May be a trustworthy result that both masks and respirators worn continuously are protective but the control group was non randomised and the study is also potentially underpowered.
4. **MacIntyre et al. 2013** [21]	Three arm cluster randomised trial: surgical masks (continuous use) versus N‐95 respirators (targeted use) versus N‐95 respirators (continuous use). *N* = 1669 healthcare workers in 19 tertiary hospitals in Beijing from emergency and respiratory wards. Masks worn continuously on shift except for targeted respirator arm where they were worn during high risk procedures only. Fit testing conducted. Adherence recorded on daily diary cards.	1. CRI: 17.1% in medical mask group versus 11.8% in targeted respirator group versus 7.2% in continuous respirator group. 2. ILI: 0.7% in medical mask group versus 0.4% in targeted respirator group versus 1.0% in continuous respirator group. 3. Laboratory‐confirmed viral respiratory infection: 3.3% in medical mask group versus 3.3% in targeted respirator group versus 2.2% in continuous respirator group. 4. Laboratory‐confirmed influenza A or B: 0.2% in medical mask group versus 0.4% in targeted respirator group versus 0.5% in continuous respirator group. 5. Laboratory‐confirmed bacterial colonisation: 21.0% in medical mask group versus 14.5% in targeted respirator group versus 9.0% in continuous respirator group. **Conclusion: ITT analysis showed significant results for CRI, Bacterial Colonisation and pooled lab‐confirmed viral or bacterial infections.**	Discussion of mechanisms primarily in relation to identifying transmission episodes (p. 963). No specific mechanism endorsed. Filtration efficiency of different types of masks also mentioned (p. 960).	The study accommodates a wide range of potential transmission mechanisms. The significant difference between some arms may be trustworthy, though there was no difference in the primary outcomes relating to viruses.
5. Radonovich et al. 2019 [22]	Pragmatic two arm cluster randomised trial: surgical masks versus N95 respirators. *N* = 1993 healthcare workers from 137 outpatient sites in the US across a range of departments for 4 influenza seasons. Masks worn when within 1.83 m of a patient with suspected or confirmed respiratory illness. Fit testing conducted annually. Independent observation to confirm adherence but incomplete (did not enter patient rooms) and via daily diary cards.	1. Laboratory‐confirmed influenza: 7.2% in the medical mask group versus 8.2% in the N‐95 respirators (difference,1.0% [95% CI, −0.5% to 2.5%]; *p *= 0.18) (adjusted OR, 1.18 [95%CI, 0.95‐1.45]). **Conclusion: No significant difference between arms.**	Yes, only aerosol and droplet mentioned. Droplet described as main mechanism (p. 830). Differing filtration efficiency noted (p. 825).	Targeted use of all masks (only when within 1.83 m of patient) may have been insufficient to capture differences between masks given possibility of aerosol transmission. Note that in light of behavioural mechanisms, targeted use relies on individuals accurately identifying risk.

^a^ Defined as symptoms of a body temperature 38°C or greater and new or worsening cough or shortness of breath.

The decision to include an arm where the N95 respirator is worn continuously engages with a number of mechanistic issues working at multiple levels. The authors offer the following mechanistic analysis (p. 963): ‘Whether targeted use of masks or respirators is protective depends on whether HCWs [health care workers] can accurately identify all episodes of risk, whether transmission occurs only during clearly identified exposures, and whether there is transmission from asymptomatic or presymptomatic individuals.’

Underlying these observations are the mechanisms of airborne transmission via infectious droplets or aerosols. Pathogens are emitted from the respiratory tract within droplets (above 100 microns in diameter which fall under the influence of gravity) and aerosols (smaller droplets less than 100 microns, which can remain suspended in air and travel to fill an indoor space) [23]. Aerosols are more efficiently filtered by respirators than surgical masks as the latter do not fit closely to the face and are made with materials of a lower filtration capacity. Note that these mechanisms of transmission operate across multiple levels of explanation, encompassing the physics of microscopic particles to human physiology and materials science in relation to filtration capacity of masks. In addition to airborne transmission, respiratory illnesses are also understood to spread through fomites (contact with contaminated objects) [24]; this is not a focus of our analysis as that transmission route is addressed via interventions such as handwashing rather than masking.

Fundamental study design choices such as which mask to wear and under what circumstances, need to take into account transmission mechanisms as they are likely to have a bearing on the results. Masking policies targeted at one of these transmission mechanisms will be ineffective or less effective if the spread of a particular illness was primarily via another route or if the details of the presumed mechanism are incorrect. For example, hospital policies appear to presuppose that the primary mechanism of transmission is via droplets and that aerosols are generated predominantly during specific procedures. However, if a particular illness is spread substantially through aerosols which spread and persist in the air for longer than the duration of the procedure, or if aerosols are produced outside of these procedures, then continuous use of a respirator will be necessary to effectively block these particles and reduce transmission [25].

As we shall see, this trial is unusual in that it considers the possibility of a wide range of transmission mechanisms in its study design, which is well‐judged particularly for a hospital setting where individuals are exposed to a wide range of respiratory illnesses with potentially varying transmission mechanisms. The continuous respirator arm is also an acknowledgement of the possibility of pre/asymptomatic transmission [26] which is relevant to mechanisms operating at a behavioural level. As the authors note, targeted use relies on health workers being able to accurately identify episodes of risk. This means not only specific procedures that generate pathogens such as intubation but potentially infectious individuals — the possibility of asymptomatic transmission suggests that there may not be cues, such as coughing or an elevated temperature, to indicate when a mask should be donned.

*Cochrane Review: Jefferson et al. 2023 ‘Physical interventions to interrupt or reduce the spread of respiratory viruses.’*

The Cochrane review [27] considers masks as well as other non‐pharmeceutical interventions such as handwashing and physical distancing. However, the most publicised results of the review were in relation to masking with the review concluding: ‘There is uncertainty about the effects of face masks. The low to moderate certainty of evidence means our confidence in the effect estimate is limited, and that the true effect may be different from the observed estimate of the effect.’ Below, the focus is on the masks versus respirators comparison where the key trial features. We shall find that the current approach which considers outcome evidence in isolation misses important aspects of study quality and compromises the rigour of evidence reviews.

Overall, the Cochrane review included 19 RCTs of masks. The data set was highly heterogenous, spanning multiple countries (Bangladesh, China, Saudi Arabia, Germany, United States, Australia, Hong Kong, Japan), settings (universities, households, healthcare settings, the Hajj pilgrimage), populations (students, healthcare workers, religious pilgrims) and evaluated various combinations of masking interventions (masking vs control; masking vs and in combination with hand washing; surgical masks vs. cloth or respirator masks) as well as some studies evaluating behavioural and educational interventions relating to masking (e.g. increased public health messaging relating to masking). Full details of the included RCTs can be found in our analysis of the trials [13] and in the list of included studies in the Cochrane review.

Table 1 analyses the five trials included in the masks vs respirators comparison in the review for: details of whether there is any discussion of mechanisms particularly transmission mechanisms; which transmission mechanism the authors appear to believe is the primary one and offers a preliminary mechanism informed analysis of how the presumed transmission mechanisms may have influenced the observed results.

Three out of the five trials [19, 20, 28], do not endorse any particular mechanism and consequently include some provision for continuous masking, given the possibility of aerosol transmission. Two of these trials (MacIntyre et al. 2011; 2015) show statistically significant differences amongst some primary outcomes. In contrast, the two remaining trials (Loeb et al. 2009; Radonovich et al. 2019) explicitly state that droplets are the primary transmission mechanism and thus have only targeted rather than continuous masking in the study design. Both of these non‐inferiority trials did not register any differences between arms. The decision to have respirators worn in a targeted manner rather than continuously appears to have had an impact on the observed results, demonstrating that the circumstances under which masks are worn matter to registering their efficacy. Consequently, consideration of mechanisms are vitally important for evaluating whether choices of study design can maximise the chances of registering a significant difference.

Additionally the meta‐analysis for the surgical masks vs respirators comparison combined the two respirator arms^1^ of the key trial, obfuscating the importance of continuous respirator masking. If the circumstances under which respirators were worn were taken into account — i.e. the results of continuous respirator use were separated from targeted use — it is plausible that the meta‐analysis would have found a significant difference.

## 2 Risk of Bias Analysis

ROB tools are primary means through which study quality is currently appraised, with quality taken to be synonymous with limiting bias. The Cochrane ROB tool for RCTs assesses bias across six domains: Random sequence generation (selection bias); Allocation concealment (selection bias); Blinding of participants and personnel (performance bias): Blinding of outcome assessment (detection bias); Incomplete outcome data (attrition bias); Selective reporting (reporting bias). Most reviewed studies had a high risk of bias for most of these domains or an unclear risk where there was insufficient data provided by the study authors.

When evaluating study quality, it is necessary to consider how studies have accounted for the relevant mechanisms in their design in addition to considering traditional domains of bias. According to the ROB analysis, Radonovich et al. 2019 is the highest quality trial scoring low risk across all domains. In contrast, MacIntyre et al. 2013 scored an unclear risk of bias for the first two domains and low risk for the remaining four. Radonovich is very similar to the key study in that it also compared surgical masks with respirators. As noted, it found no significant difference between the arms, plausibly because it did not have a continuous respirator use arm. The authors state: “Respiratory viruses are primarily transmitted by large droplets. Because a fraction of respiratory viruses may be transmitted by aerosol, N95 respirators have been presumed to provide better protection than medical masks against viral respiratory infections in health care settings”, suggesting that a continuous respirator arm was deemed unnecessary as the main transmission mechanism was assumed to be droplets. The contrast between how the two studies accounted for a range of transmission mechanisms in their study design is not reflected in the ROB analysis, thereby missing an important indicator of study quality.

The way in which endorsement of different transmissions mechanisms is closely related to study design decisions, i.e. whether to include a continuous respirator arm, invites reflection on the studies which make no explicit comments about transmission mechanisms but do not include a continuous respirator arm. Despite not explicitly endorsing any particular mechanism, the decision not to include a continuous respirator arm can be read as a tacit claim that the primary mode of transmission is likely to be droplets. As such, basic design choices, such as the conditions under which an intervention is deployed, involves subscribing to some mechanistic account over another even when there is no explicit endorsement of a particular mechanistic account.

Mechanistic analysis can also assist with the standard ROB domains as can be seen in the blinding of participants and personnel domain. The key study was described as low risk for blinding despite it being impossible to blind the type of mask. The corresponding author of the review clarified (in email communication) that the “low” rating was given because the study authors had stated in‐text that blinding would not affect the primary outcome as it was additionally confirmed with lab‐testing. In order to evaluate whether a reason supplied by the authors of study justifies a particular risk rating, it is necessary to consider the relevant mechanisms. In this case, there could be multiple mechanisms through which knowledge of the intervention could affect the outcome. To consider a mechanism operating at the behavioural level, continuous respirator use may have made participants more cognisant of risks and they may have adopted other measures which limited their exposure, such as avoiding mass gatherings outside of work. Similar mechanistic analysis is required to avoid inconsistency; many of the other trials confirmed the primary outcome with lab testing but received a high risk score for the blinding domain^2^. If lab testing mitigates the lack of blinding then the same mechanisms should hold across the trials and they all should have been assigned the same ROB score for that domain.

### 2.1 GRADE Analysis

While ROB analysis appraises individual study quality, GRADE evaluates the quality of the entire body of evidence for a particular question or comparison considered by the review such as the masks versus respirators comparison. GRADE considers five domains: study limitations, consistency of effect, imprecision, indirectness, and publication bias. The evidence for each outcome relating to the review question or comparison is appraised according to these domains and upgraded or downgraded (usually the latter) according to limitations in the domains. Just as aspects of study quality related to how well a study accounts for relevant mechanisms is neglected in the ROB analysis, mechanistic analysis does not feature in GRADE appraisals, meaning that bodies of evidence are not scrutinised for how well they take account of relevant mechanisms. The first domain of study limitations appears most appropriate for including mechanistic analysis, however it is currently synonymous with ROB analysis.

Mechanistic analysis is also vital in supporting current GRADE appraisals. For example, in the Cochrane review, the absence of mechanism‐informed analysis has replicated the same issues noted in the ROB analysis. Lack of blinding is accepted as a study limitation warranting downgrading a level in the case of the three primary outcomes involving self‐reporting of symptoms (clinical respiratory illness; influenza like‐illness and adverse events) but not laboratory confirmed illness. Thus, the questionable rationale for labelling Radonovich et al. 2019 and MacIntyre et al. 2013 as low risk in the ROB analysis was also accepted in the GRADE analysis when downgrading with no further evaluation. Mechanistic analysis would have determined whether such reasoning was sound.

### 2.2 Case Study 2: Psychotherapy for Treatment Resistant Depression

*Key Trial: Fonagy et al. 2015 Pragmatic randomised controlled trial of long‐term psychoanalytic psychotherapy for treatment‐resistant depression: the Tavistock Adult Depression Study.*

Depression is one of the most prevalent mental health disorders, with the WHO reporting that globally, an estimated 5.7% of adults suffer from depression [30]. Recommended first line treatment, or treatment as usual (TAU), includes pharmaceuticals such as antidepressants and psychological therapy. A depressive disorder is described as treatment resistant when it does not respond to TAU. Unlike many psychiatric conditions, there is no definitive definition of treatment resistant depression (TRD) in the Diagnostic and Statistical Manual of Disorders (DSM) or the International Classification of Diseases (ICD). Consequently, TRD is characterised in both trials and reviews in terms of the inclusion criteria.

Fonagy et al. 2015 was a three arm, pragmatic RCT comparing TAU with TAU supplemented by long‐term psychoanalytic psychotherapy for TRD. TRD was characterised in terms of: DSM‐IV diagnosis of Major Depressive Disorder; > 2 years of current depressive episode; Hamilton Depression Rating ≥ 14 score; Beck Depression Inventory score ≥ 21; two failed previous treatments for Major Depressive Disorder, one of which had to be with an antidepressant.

Patients (*n* = 129) were recruited from primary care in the UK and those in the active group were provided with 60 (50 min) weekly sessions of psychoanalytic psychotherapy over 18 months. The primary outcome measure was the clinician rated Hamilton Depression Rating Scale (HAM‐D). Data was collected at baseline, 6, 12, 18 months over the course of treatment, and at 24, 30, 42 months during follow‐up.

A notable feature of this trial is its extended length particularly during follow‐up. The length is related to mechanistic analysis of: how depression might be maintained in individuals with TRD, the mechanisms through which LTPP may address these maintaining factors and the time‐scale on which these various mechanisms may act. The authors state: ‘The theory employed in LTPP [long‐term psychoanalytic psychotherapy] assumes that, in patients with treatment‐resistant depression, problems with psychosocial functioning impair help‐seeking and illness‐combating behaviours, and may also have an emotional impact upon health care/service providers in a way that affects the care they offer. LTPP enables these patients to gradually internalise a psychological capacity to relate to pathogenic personal experiences, memories, feelings, beliefs and relationships in a reflective, yet also more active, manner [31] (p. 313)’.

The authors' statements suggest that the therapeutic mechanisms of psychoanalysis can be counteracted by some of the mechanisms maintaining the condition (e.g. interpersonal difficulties with care providers) requiring a longer time‐scale for resolving an entrenched condition such as TRD. Wider mechanistic research into psychotherapy also suggests that the time‐scale on which therapeutic mechanisms act may be longer than expected and non‐linear with an initial reluctance to ‘open up’ and potential for therapeutic rupture [31, 32]. Consequently, as the authors state, ‘End‐of treatment evaluations or short follow‐ups may miss the emergence of delayed therapeutic benefit’ (p. 312).

There was no significant difference in the mean primary outcome between the groups at the end of treatment (at 18 months: 16.4 in the psychoanalysis group vs. 17.9 in TAU, *p* > 0.05). However, there were significant differences at each stage of follow‐up (at 42 months: 15.9 in the psychoanalysis group vs. 20.1 in the TAU group, *p* < 0.001). Additionally, at the end of 42 months, 40% of the psychoanalysis group no longer met the criteria for Major Depressive Disorder.

*Cochrane Review: Ijaz* et al. *2018 Psychological therapies for treatment‐resistant depression in adults*.

The review examined the outcomes of supplementing TAU with psychological therapy for TRD, with the condition being characterised primarily as non‐response to at least 4 weeks of antidepressant medication (p. 9). The review concluded: ‘Moderate‐quality evidence shows that psychotherapy added to usual care (with antidepressants) is beneficial for depressive symptoms and for response and remission rates over the short term for patients with TRD. Medium‐ and long‐term effects seem similarly beneficial, although most evidence was derived from a single large trial. Psychotherapy added to usual care seems as acceptable as usual care alone.’ (p. 2)

The review included six trials, most of which had small sample sizes, barring one large CBT trial. Two trials were based in the UK, the others in the US, Japan, Canada and Brazil. The trials examined a number of therapies: individual CBT in half, with three trials examining group dialectical behaviour therapy, interpersonal therapy, short term psychodynamic therapy. Table 2 offers a summary of the included studies and a preliminary mechanistic analysis.

**Table 2:** Psychological therapy trials.

	Study design	Characterisation of TRD^a^	Primary outcomes and results^b^	Mechanism informed analysis
1. Harley et al. 2008 [33]	Pilot of group Dialectical Behaviour Therapy versus Wait‐list control^c^ *N* = 19 (referred by outpatient psychiatric services in the UK) Dose: 16 sessions (weekly, 1.5 h, with additional tasks between sessions) Follow‐up: 6 months	6 weeks on a stable dose of antidepressants; diagnosis of Major Depressive Disorder (MDD). Psychosocial impairment noted as maintaining mechanism and important target for therapy (p. 136).	1. Hamilton Depression Rating Scale (HAM‐D) at baseline and end of treatment: 11.3 therapy vs 17.11 (Cohen's d = 1.45, *p* < 0.05) at week 16. No follow‐up data collected. 2. Beck Depression Inventory (BDI) at baseline, end of treatment and follow‐up: 15.1 therapy versus 25.89 (Cohen's d = 1.31, *p* < 0.01) at week 16. 15.2 at follow‐up (*n* = 8; t‐test= 0.624, not significant). **Conclusion: Significant differences across both primary outcomes at end of treatment. No significant difference between end of treatment and follow‐up.**	6 weeks on medication is an inadequate characterisation of TRD. 6 months follow‐up is unlikely to be sufficient to capture stability of outcomes. Note that despite identifying psychosocial mechanisms as being significant, the secondary outcome measures which tracked social and functioning changes failed to find a significant difference with the control. The authors suggest that the dose and duration of the trial may have been too brief to accomplish those changes (p. 142).
2. Wiles et al. 2008 [34]	Pilot of CBT versus TAUN = 25 (recruited by GP in UK)Dose: 12–20 sessions (median 9.5 sessions) Follow‐up: None. Data collected at baseline and 4 months post randomisation only.	6 weeks on a stable dose of antidepressants; diagnosis of MDD on ICD‐10; BDI‐II ≥ 15Note that individuals with depression diagnosis for over 5 years were excluded. Definition of TRD compared to NICE definition which is based on failure to respond to two sequential doses. Authors claim that their definition is more clinically useful and NICE's is not backed by RCT data (p. 31)	1. BDI (baseline, 4 months post randomisation): No raw data provided, only results of adjusted regression analysis. Authors state that mean BDI in CBT group was 9 points lower than in TAU, with 8 out of 14 patients showing a 50% reduction in symptoms from baseline. **Conclusion: No significance test reported.**	6 weeks on medication is an inadequate characterisation of TRD. No follow‐up so no ability to track stability of outcomes.
3. Nakagawa et al. 2017 [35]	Pragmatic RCT of CBT with TAU vs TAU alone *N* = 80 (one university and one psychiatric hospital in Japan) Dose: 16 sessions (weekly, 50 min, 4 additional available) Follow‐up: 3, 6, 12 months	8 weeks on a stable dose of antidepressants; diagnosis of MDD on DSM‐IV; Maudsley Staging Criteria for TRD score≥ 3; HAM‐D 17 (GRID) ≥ 16Maudsley staging criteria discussed in detail (p. 1127)	1. HAM‐D collected at baseline, 8 weeks, end of treatment. Follow up at 3, 6, 12 months: Least squares mean changes ‐12.7 in CBT group versus ‐7.4 in TAU (between group mean difference ‐5.4, *p* < 0.001) at end of treatment. Least squares mean changes ‐15.4 in CBT group vs ‐11.0 in TAU (between group mean difference ‐4.4, *p* = 0.002) at end of treatment. **Conclusion: Significant difference between groups at end of treatment. No significant difference between end of treatment and follow‐up.**	The sample included participants where 60% had undergone > 3 courses of antidepressants suggesting the characterisation of TRD was satisfactory (p. 1133). The length of antidepressant treatment was only 8 weeks but was supplemented by the Maudsley staging criteria (note however that it tracks the lower end of the severity spectrum). 12 months follow‐up may be sufficient to capture stability of outcomes.
4. Souza et al. 2016 [36]	Pragmatic RCT of Interpersonal therapy (IPT) with TAU versus TAU alone *N* = 40 (tertiary outpatient service in Brazil)Dose: 16 sessions (weekly, 40 min, mean sessions completed 11.53) Follow‐up: None/unclear. Data collected at baseline and 8, 12, 19, 24 weeks post randomisation	4 weeks on a stable dose of antidepressants; diagnosis of MDD on DSM‐IV. Notes that there is controversy about TRD definition and that longer term psychotherapy is indicated for TRD (p. 378)	1. HAM‐D 17: 14.0 at week 24 for IPT versus 13.9 for TAU. The mean difference from baseline was 5.86 for IPT versus 4.57 for TAU. No significant difference between groups at any time in the linear mixed model analysis. **Conclusion: No significant difference between groups. No follow‐up data collected.**	4 weeks on medication is an inadequate characterisation of TRD. No follow‐up so no ability to track stability of outcomes.
5. Wiles et al. 2016 [37, 38]	Pragmatic RCT of CBT with TAU versus TAU alone *N* = 469 (recruited from 73 GP's in the UK)Dose: 12 sessions (50‐60 min, extra 6 available, median 12 sessions completed by 12 months) Follow‐up: 40 months post treatment (published separately)	6 weeks on a stable dose of antidepressants; meets ICD‐10 criteria for depressive episode; BDI ≥ 16 Cognitive rather than behavioural mechanisms noted as an important target for persistent depression (p. 376, 377, 382). Definition of TRS noted as more clinically useful (p. 381)	1. BDI (collected at 3, 6, 9, 12 months post randomisation and approx 40 months after treatment): At 12 months mean BDI score 17.0 for CBT and 21.7 for TAU (Adjusted difference in means ‐5.1, *p* < 0.001). At 40 months (*n* = 248) mean BDI score 19.2 for CBT and 23.4 for TAU (Adjusted difference in means in repeated measures analysis ‐4.7, *p* < 0.001) **Conclusion: Significant differences across both primary outcomes at end of treatment. No significant difference between end of treatment and follow‐up.**	The sample included participants who had ≥ 5 episodes of depression (50% in CBT and 55% in TAU) and whose current episode was ≥ 12 months (58% in CBT and 60% in TAU) suggesting that the characterisation of TRD was satisfactory. The length of antidepressant treatment was only 8 weeks but criteria of BDI ≥ 16 may have contributed to capturing a suitable sample. Though note that this may track chronic depression rather than TRD. No data collected on previous failed antidepressant treatments, only whether antidepressants had ever been prescribed and how long current prescription had been taken for.46 months follow up is likely to track stability of outcomes.
6. Town et al. 2017 [39]	Intensive Short‐term psychodynamic psychotherapy (ISTDP) versus TAUN = 60 (secondary care outpatient community mental health services in Canada) Dose: 20 sessions (2–3 h initial case formulation then weekly hourly session, mean sessions = 16.1) Follow‐up: None/unclear. Data collected at baseline, 3 months and 6 months post randomisation. Note that longer follow‐up data for this study was published later but was not available at the time of the Cochrane review.	Stable dose of antidepressants for ≥ 6 weeks; ≥ 1 trial of antidepressants at adequate dose; current depressive episode ≥ 6 weeks; HAM‐D ≥ 16; Thase and Rush 5‐StageModel and the Massachusetts GeneralHospital Staging Model (p. 16) used to support characterisation of TRD. Mechanism maintaining depression and therapeutic mechanism described in terms of emotional factors (p. 16). Pharmaceutical mechanisms also discussed (p. 17) in terms of noting lack of washout phase between antidepressant treatments. Possibility of some patients requiring long term psychotherapy if symptom change is not sustained after a brief intervention noted (p. 23).	1. HAM‐D: Estimated mean difference at 6 months post randomisation 12.405 for ISTDP group versus 18.675 for TAU (Cohen's d = 0.748, *p* < 0.05) **Conclusion: Significant difference between groups. Follow‐up data not reported in this study.**	The sample included participants (63.8%) who had failed at least two pharmacotherapy trialsfrom two distinct antidepressant classes previously suggesting that the characterisation of TRD was satisfactory (p. 22). The length of antidepressant treatment was only 6 weeks but was supplemented by additional criteria which may have contributed to capturing a suitable sample. No follow‐up reported so no ability to track stability of outcomes in the current study.

^a^ Note that in all cases characterisation of TRD in inclusion criteria was accompanied by the exclusion of other comorbid psychiatric conditions.

^b^ Higher scores indicate greater number or severity of symptoms on outcome measures such as HAM‐D (clinician‐rated) and BDI (self‐report). Cohen's d is an indication of effect size with values interpreted as: small (d > 0.2), moderate (d > 0.5), and large effect sizes (d > 0.8).

^c^ Wait‐list refers to a control condition where participants receive the intervention after the trial is concluded.

Fonagy et al. 2015 alongside another well known large‐scale trial for TRD – the STAR‐D trial [40] – were excluded by the review as they did not match the inclusion criteria, specifically the characterisation of TRD adopted. Notably, in the reasons for exclusion table, the authors make an additional comment with regard to the key trial: ‘dose and duration of treatment not a consideration for inclusion’ (p. 45). For a review of TRD, it is implausible that the dose and duration of treatment is irrelevant because the mechanisms maintaining the condition as well as the therapeutic mechanisms entail reflection on time‐scales, that is, how much therapy and for how long is required to demonstrate an effect.

In the psychological therapy literature, mechanistic accounts vary between therapeutic modalities and are evidenced to varying degrees. Though Fonagy et al. 2015 offers a mechanistic analysis based on psychoanalytic theory, other schools of therapy, including CBT which is recognised for its evidence‐base, also provide mechanistic reasoning to support the idea that conditions such as TRD require longer term therapy to address deeper rooted problems. For example, schema therapy [41], was developed from CBT to address more fundamental cognitive schemas as it hypothesised that they are responsible for maintaining long term conditions such as personality disorders and TRD. It is understood that to address schemas adequately, more sessions over a longer period of time is required. In contrast to the key trial, most of the reviewed RCTs included approximately 16 sessions of therapy. The broad agreement on the dosage and timescales on which the mechanisms act between different types of therapy (despite offering different accounts of the specific therapeutic mechanisms) demonstrates the utility of mechanistic analysis even when there is no consensus.

Not only are the inclusion criteria at odds with mechanistic understandings from a variety of therapeutic models for how TRD is maintained and treated, it is additionally at odds with the mechanisms presupposed in clinical understandings of the condition as well as mechanisms of drug action. For example, the characterisation of TRD does not tally with the NICE understanding of TRD [42] or the Maudsley prescribing guide [43]. Both cite non‐response to two courses of antidepressants, as does the DSM and the ICD. Furthermore, common antidepressants such as selective serotonin inhibitors do not show efficacy immediately or require adjustment in dosage [44] – 4 weeks and one course of treatment is too short a time period to determine that the depression is treatment resistant.

A large number of the studies excluded by the review were based on this characterisation of TRD which fails to account for the mechanisms relevant to the condition and the time scales on which they act. Half of the included studies used a similar characterisation of TRD [33, 34, 36]. The others supplemented length of time on an antidepressant with additional criteria such as cutoff scores on the outcome measures and the Maudsley guidelines. Those studies appeared to capture a sample more representative of the condition with participants who had more complex and severe cases of depressive disorder as well as multiple failed courses of antidepressants as seen in Table 2. However, it is possible that studies which did not explicitly enquire into previous courses of antidepressants may be capturing chronic rather than treatment resistant depression.

### 2.3 Risk of Bias and GRADE Analysis

The following offers a briefer analysis of quality appraisals as the Cochrane review does not include the key trial, barring the possibility of a comparative analysis as in the previous case study.

Even with standard ROB and GRADE analysis, consideration of mechanisms is necessary. Using the Cochrane ROB tool, the review states that across most domains the six RCTs show low risk of bias with high risk of bias for participant blinding and blinding of outcome assessment. This is plausible given the difficulty of blinding psychological interventions and many of the outcome measures using self‐report. Blinding becomes an issue at the level of the GRADE analysis. When downgrading for the study limitation domain, only lack of blinding in outcome assessment is cited. No further explanation is offered for why relying on self‐report outcomes is more concerning than an unblinded intervention. In explaining and making transparent why only one of these is sufficient to warrant downgrading a level, it seems plausible that mechanistic analysis will be required.

When considering the mechanisms involved in maintaining and treating TRD, the time scales matter. Consequently, the number of sessions and length of follow‐up are indicators of the quality of the trial, which are not captured in a standard ROB analysis. There is some acknowledgement of the need to track long‐term outcomes in the review, with a separate analysis of long‐term outcomes from 12 months onward. As shown in Table 2, of the six trials, only two have a long follow‐up period, with many simply tracking outcomes during the trial or shortly afterwards. There is no indication that the longer studies were considered to be of higher quality.

Overall, the inadequate characterisation of TRD calls into question the conclusions of the review as it seems implausible that the included studies are able to address the research question. The GRADE domain of indirectness in theory deals with this question. However currently it is understood more narrowly in terms of whether and to what extent the assembled evidence deviates from the PICO (Population, Intervention, Comparison, Outcome) framing of the research question rather than in light of a deeper mechanistic analysis.

### 2.4 Wider Implications

The preceding analysis has demonstrated the importance of mechanism‐informed study design and evidence synthesis. One of the main limitations of the current study is that it only examines two cases. The case studies were chosen, in part, to demonstrate the range of areas where mechanistic analysis is useful. It seems plausible that other cases will highlight additional areas. Mechanistic analysis appears to be tailored to each case. For instance, with TRD, it was necessary to reflect on the inclusion criteria as there is no settled definition of the condition which is atypical (though in the mental health field, there is an issue of how to define and demarcate psychosocial interventions which is more widespread).

Another limitation is that the present study does not offer a comprehensive or formalised account of a number of pressing issues such as: which mechanisms and at which levels, should be considered when appraising a study or evidence review, how to evaluate evidence for mechanistic accounts and how to resolve contested evidence for mechanisms. The study has instead offered a fine‐grained critique of the status quo, focusing on a small number of mechanisms to show in some detail how evaluating outcome research in isolation from mechanistic research results in insufficient consideration of study quality and compromises to the rigour of evidence reviews. Future research should focus on these areas in more detail; some preliminary suggestions are offered below.

Despite these limitations, it should be apparent that mechanistic analysis is deeply entangled with traditional outcome research, as it is not possible to avoid thinking about mechanisms when making basic study design choices or considering the parameters of an evidence synthesis. Even where there is no explicit endorsement of a particular mechanistic account, certain basic design choices in studies and in review parameters entails tacitly endorsing particular mechanistic accounts over others. As such, the way in which mechanistic considerations have shaped a study or a review must be reported transparently – perhaps in expanded versions of the current SPIRIT guidance and Cochrane's mandatory ‘How the intervention might work’ sections – and be accounted for in evaluations of quality. In quality appraisals, mechanistic analysis is fruitful in supplementing traditional GRADE and ROB analyses and other identifying aspects of study quality. The preceding analysis entails some wider reflections and recommendations.

### 2.5 Expanded Notions of Quality

Currently, the quality of studies and bodies of evidence are understood in fairly limited ways. For example at the level of individual studies, ROB analysis focuses on set criteria such as whether the study has successfully blinded and randomised participants rather than evaluating the extent to which the study has accounted for relevant mechanisms. In addressing this problem, it may be possible to supplement the current system of quality appraisal with consideration of mechanisms. For instance, ROB analysis could include broader understanding of study quality with mechanistic analysis. Additionally, in the TRD review, the issues with the characterisation of the condition could be understood as a problem to be considered in the indirectness GRADE domain. Though this domain is currently understood in a far more narrow sense, the intent is to evaluate the extent to which the available body of evidence is able to answer the research question.

Though this would be welcome progress, the preceding analysis highlights the necessity of not just supplementing but interrogating our notions of quality. The current approach conceptualises study and evidence quality primarily in terms of controlling various forms of bias and confounding. As such quality is understood in a somewhat negative sense, where quality is related to how well certain issues have been avoided. Mechanistic analysis highlights a more expansive, positive dimension of quality, namely the extent to which it has maximised the chances of registering a true result.

Above, we have considered only a small cluster of mechanisms and the way in which considering them in the study design appears to have an effect on the results. For instance, continuous use of respirators was necessary to register a significant difference given transmission mechanisms as was longer term‐outcome tracking in the case TRD given the timescales of the mechanisms. Another set of relevant mechanisms, further illustrates the need to account for mechanisms. The background prevalence of an infectious disease such as a respiratory illness, may determine the outcome of the study because when the prevalence is high, it will be easier to register an effect. Running masking trials during particular periods such as the winter influenza period and having a sufficiently long monitoring and testing window would mitigate the effects of fluctuations in prevalence. RCTs for masking were all conducted during local flu seasons. However, many of the studies still had issues with background prevalence, in most cases because the monitoring and testing window was too brief as noted in our wider analysis of the data set [13]. The primary outcomes of the key trial also suggest that it has gotten unlucky despite accounting for these issues in the study design as it registered significant differences in outcomes related to bacterial infection but not viral^3^. This shows the importance of good study design – even when you optimise your study design in light of mechanisms, you may still get unlucky. But your odds are better.

Standard ROB and GRADE analyses are often presented as ‘objective’ given their highly formalised nature. However, our analysis of the wider set of masking evidence reviews [14] demonstrates a wide range of judgements of the same studies across different reviews. This inconsistency should be unsurprising as the preceding analysis has demonstrated inconsistency even within the same review, due to insufficient consideration of mechanisms. It is unclear whether greater consideration of mechanisms will bring consistency and consensus across review teams, but making the mechanistic assumptions underpinning these judgements more transparent will at least highlight the points of disagreement which are currently opaque.

### 2.6 Topic Expertise Alongside Methodological Expertise

Systematic review is often viewed as an exercise solely in data extraction and quantitative analysis, requiring expertise only in evidence synthesis methodology with review teams often lacking topic‐specific expertise. Though this is not the case for the Cochrane reviews, our analysis of the review teams for other masking studies revealed this gap as did an ethnographic study of systematic reviewers [14, 45]. Review teams who wish to undertake more rigorous mechanistic analysis will need a greater number of topic experts familiar with the primary literature in addition to methodological experts. Though this appears labour intensive, AI supported methods, such as those developed in our wider project may be fruitful in a supportive role. In the wider project, Generative Pre‐trained Transformers were built by topic experts who had identified the most relevant mechanisms, to analyse the available outcome research, particularly the ways in which observed results could be accounted for by these mechanisms [46]. This is only one such approach and it seems plausible that the new technologies will facilitate mechanistically informed synthesis and study appraisal in other ways.

### 2.7 Alternate Study Designs

RCTs are now considered the gold‐standard study design with other designs automatically downgraded two levels in the GRADE analysis. Additionally, appraisals of quality, both at the level of individual studies and the bodies of evidence rely on a tacit idea of the ideal RCT. For example, studies are evaluated based on whether they are blinded in various aspects. There remain questions about whether RCTs are truly the gold standard for all cases. Particularly when the standards of an ideal RCT simply cannot be met as in both the cases considered where blinding of the intervention is impossible. In engaging with mechanistic analysis, we will be better positioned to identify alternate study designs. For example, with entrenched conditions such as TRD, we saw that it was necessary to track outcomes over a longer period. However, it is expensive and difficult to run RCTs for an extended period making practice based research studies – where outcomes are tracked alongside routine service delivery – an attractive alternative. Naturally there are impediments to utilising other study designs. For instance, the risk of many types of bias is greater and there are difficulties in aggregating across studies. However, it must be considered an open question whether other study designs will be better suited for certain research questions in light of mechanistic considerations.

### 2.8 A Unified Research Programme

The current bifurcation of outcome research and mechanistic research has yielded poor results. Given the enormous resources that go into outcome research, it is troubling that many systematic reviews and meta‐analyses are inconclusive, often because the available evidence is of low quality [47, 48, 49]. Given that appraising study quality involves mechanistic analysis, bringing together outcome and mechanistic research is likely to improve the current situation. On top of evaluating study quality and making many areas of evidence synthesis more transparent and rigorous, mechanistic analysis will also offer a diagnosis of why the current bodies of evidence are lacking and provide suggestions for improvements to study design for future research rather than proliferating low‐quality trials as with the status quo. Despite the artificial separation, these two streams of research are in fact part of one research programme.

## Ethics Statement

The author has nothing to report.

## Conflicts of Interest

The author declares no conflicts of interest.

## Data Availability

The author has nothing to report.

## Footnotes

1 This has been confirmed by the statistician on the review team (pers. comm).

2 Three other masking studies received a low ROB score for the blinding to the intervention domain (Barasheed et al. 2014, Ide et al. 2016, Radonovich et al. 2019). Barasheed and Ide are plausible. The former was conducted at Hajj where masks are already distributed by local health agencies and questions about masking are commonplace (the intervention was supervised mask use rather than mask use simpliciter) [29]. Ide considered the treatment of masks with a compound that could not be detected. However, the low risk of bias assigned to Radonovich is likely mistaken for the reasons outlined above.

3 Note that this is one possible explanation for the observed results. The vital point is that many explanations for an observed pattern of results, e.g. why trials with a continuous respirator arm or ones with a longer testing window show a different pattern of results, appeals to counterfactual reasoning which draws on mechanistic analysis.

## References

[1] T. Greenhalgh , D. Fisman , D. J. Cane , M. Oliver , and C. R. Macintyre , “Adapt or Die: How the Pandemic Made the Shift From EBM to EBM+ More Urgent,” BMJ Evidence‐Based Medicine 27, no. 5 (2022): 253–260, 10.1136/bmjebm-2022-111952. PMC9510422 35853682

[2] Centre for Evidence‐Based Medicine (CEBM) . University of Oxford [Internet]. OCEBM Levels of Evidence, https://www.cebm.ox.ac.uk/resources/levels-of-evidence/ocebm-levels-of-evidence.

[3] EQUATOR Network , SPIRIT 2025 Expanded Checklist Detailing Items to Address in Randomized Trial Protocols, Based on Explanation and Elaboration Document, https://www.consort-spirit.org/_files/ugd/b5740e_ffb985d849cc41afbe66d58babc8653f.pdf.

[4] J. Thomas , M. Petticrew , J. Noyes , et al., Chapter 17: Intervention Complexity. In: Higgins J , Thomas J , Chandler J , Cumpston M , Li T , Page, editors. Cochrane Handbook for Systematic Reviews of Interventions [version 6.5], cochrane.org/handbook.

[5] P. M. Illari and J. Williamson , “What Is a Mechanism? Thinking About Mechanisms Across the Sciences,” European Journal for Philosophy of Science 2, no. 1 (2012): 119–135, 10.1007/s13194-011-0038-2.

[6] W. C. Salmon , Causality and Explanation (Oxford University Press, 1998), 448.

[7] Jeremy Howick , The Philosophy of Evidence‐based Medicine (Wiley‐Blackwell, BMJ Books, 2011), 245.

[8] V. P. Parkkinen , C. Wallmann , M. Wilde , et al., Evaluating Evidence of Mechanisms in Medicine: Principles and Procedures. Springer, 2018), 31314227, http://www.ncbi.nlm.nih.gov/books/NBK543869/. 31314227

[9] S. Ioannidis and S. Psillos , Mechanisms in Science [Internet] (Cambridge University Press, 2024), https://www.cambridgebookshop.co.uk/products/mechanisms-in-science.

[10] T. Greenhalgh , R. Helm , L. Poliseli , S. Ratnayake , A. Trofimov , and J. Williamson , “Protocol: A Systematic Review+ (SR+) to Combine Associative and Mechanistic Evidence on the Efficacy of Face Masks in Reducing Transmission of Respiratory Diseases,” Systematic Reviews 14, no. 1 (2025): 226, 10.1186/s13643-025-02973-2. PMC12625625 41250228

[11] R. K. Helm , T. Greenhalgh , J. Williamson , and A. Trofimov , “When Trial Interventions are Likely to Reduce the Impact of Rape Myths on Juror Decisions: A Mechanism‐Informed Evidence Synthesis,” Journal of Criminal Justice 104 (2026): 102634, 10.1016/j.jcrimjus.2026.102634.

[12] S. Ratnayake , “A Philosopher Asks Whether Therapy Should be Evaluated Like Drugs,” British Journal of Psychiatry, ahead of print, June 1, 2026, 10.1192/bjp.2026.10674. 42219968

[13] T. Greenhalgh , S. Ratnayake , R. Helm , L. Poliseli , and J. Williamson , “The Randomised Controlled Trial as Cultural Product: An Illustrative Review Using Mask Efficacy Trials,” SSM ‐ Qualitative Research in Health 9 (2026): 100772, 10.1016/j.ssmqr.2026.100772.

[14] T. Greenhalgh , S. Ratnayake , R. Helm , L. Poliseli , and J. Williamson , “Synthesis Challenges in Complex Evidence: A Critical Analysis of Systematic Reviews of Face Mask Efficacy,” Research Synthesis Methods 17, no. 4 (2026): 714–733, 10.1017/rsm.2026.10072. PMC13311340 41645791

[15] R. M. Jones and D. Rempel , “Standards for Surgical Respirators and Masks: Relevance for Protecting Healthcare Workers and the Public During Pandemics,” Annals of Work Exposures and Health 65, no. 5 (2021): 495–504, 10.1093/annweh/wxab008. PMC8135753 33942848

[16] J. Schmitt and J. Wang , “A Critical Review on the Role of Leakages in the Facemask Protection Against SARS‐CoV‐2 Infection With Consideration of Vaccination and Virus Variants,” Indoor Air 32, no. 10 (2022): e13127, 10.1111/ina.13127. PMC9828278 36305058

[17] G. Bagheri , B. Thiede , B. Hejazi , O. Schlenczek , and E. Bodenschatz , “An Upper Bound on One‐To‐One Exposure to Infectious Human Respiratory Particles,” Proceedings of the National Academy of Sciences 118, no. 49 (2021 Dec 7): e2110117118, 10.1073/pnas.2110117118. PMC8670465 34857639

[18] M. Loeb , N. Dafoe , J. Mahony , et al., “Surgical Mask vs N95 Respirator for Preventing Influenza Among Health Care Workers: A Randomized Trial,” Journal of the American Medical Association 302, no. 17 (2009): 1865–1871, 10.1001/jama.2009.1466. 19797474

[19] C. R. MacIntyre , S. Cauchemez , D. E. Dwyer , et al., “Face Mask Use and Control of Respiratory Virus Transmission in Households,” Emerging Infectious Diseases 15, no. 2 (2009): 233–241, 10.3201/eid1502.081167. PMC2662657 19193267

[20] C. R. MacIntyre , Q. Wang , S. Cauchemez , et al., “A Cluster Randomized Clinical Trial Comparing Fit‐Tested and Non‐Fit‐Tested N95 Respirators to Medical Masks to Prevent Respiratory Virus Infection in Health Care Workers,” Influenza and Other Respiratory Viruses 5, no. 3 (2011): 170–179, 10.1111/j.1750-2659.2011.00198.x. PMC4941587 21477136

[21] C. R. MacIntyre , Q. Wang , H. Seale , et al., “A Randomized Clinical Trial of Three Options for N95 Respirators and Medical Masks in Health Workers,” American Journal of Respiratory and Critical Care Medicine 187, no. 9 (2013): 960–966, 10.1164/rccm.201207-1164OC. 23413265

[22] L. J. Radonovich , M. S. Simberkoff , M. T. Bessesen , et al., “N95 Respirators vs Medical Masks for Preventing Influenza Among Health Care Personnel: A Randomized Clinical Trial,” Journal of the American Medical Association 322, no. 9 (2019): 824–833, 10.1001/jama.2019.11645. PMC6724169 31479137

[23] C. C. Wang , K. A. Prather , J. Sznitman , et al., “Airborne Transmission of Respiratory Viruses,” Science 373, no. 6558 (2021): eabd9149, 10.1126/science.abd9149. PMC8721651 34446582

[24] N. H. L. Leung , “Transmissibility and Transmission of Respiratory Viruses,” Nature Reviews Microbiology 19, no. 8 (2021): 528–545, 10.1038/s41579-021-00535-6. PMC7982882 33753932

[25] T. Greenhalgh , C. R. MacIntyre , M. G. Baker , et al., “Masks and Respirators for Prevention of Respiratory Infections: A State of the Science Review,” Clinical Microbiology Reviews 37, no. 2 (2024): e0012423, 10.1128/cmr.00124-23. PMC11326136 38775460

[26] N. H. L. Leung , C. Xu , D. K. M. Ip , and B. J. Cowling , “Review Article: The Fraction of Influenza Virus Infections That are Asymptomatic,” Epidemiology 26, no. 6 (2015): 862–872, 10.1097/EDE.0000000000000340. PMC4586318 26133025

[27] Jefferson , T. Dooley , L. Ferroni , et al., Physical Interventions to Interrupt or Reduce the Spread of Respiratory Viruses ‐ Jefferson, T ‐ 2023 Cochrane Library, https://www.cochranelibrary.com/cdsr/doi/10.1002/14651858.CD006207.pub6/full. 10.1002/14651858.CD006207.pub6 PMC9885521 36715243

[28] C. R. MacIntyre , H. Seale , T. C. Dung , et al., “A Cluster Randomised Trial of Cloth Masks Compared With Medical Masks in Healthcare Workers,” BMJ Open 5, no. 4 (2015): e006577, 10.1136/bmjopen-2014-006577. PMC4420971 25903751

[29] O. Barasheed , N. Almasri , A. M. Badahdah , et al., “Pilot Randomised Controlled Trial to Test Effectiveness of Facemasks in Preventing Influenza‐Like Illness Transmission Among Australian Hajj Pilgrims in 2011,” Infectious Disorders ‐ Drug Targets 14, no. 2 (2014): 110–116, 10.2174/1871526514666141021112855. 25336079

[30] Depressive Disorder (depression) [Internet] , https://www.who.int/news-room/fact-sheets/detail/depression.

[31] C. Talbot , R. Ostiguy‐Pion , E. Painchaud , C. Lafrance , and J. Descôteaux , “Detecting Alliance Ruptures: The Effects of the Therapist's Experience, Attachment, Empathy and Countertransference Management Skills,” Research in Psychotherapy (Milano) 22, no. 1 (2019): 325, 10.4081/ripppo.2019.325. PMC7451395 32913775

[32] G. S. Kleiven , A. Hjeltnes , M. Råbu , and C. Moltu , “Opening Up: Clients' Inner Struggles in the Initial Phase of Therapy,” Frontiers in Psychology 11 (2020): 591146, 10.3389/fpsyg.2020.591146. PMC7769763 33384643

[33] R. Harley , S. Sprich , S. Safren , M. Jacobo , and M. Fava , “Adaptation of Dialectical Behavior Therapy Skills Training Group for Treatment‐Resistant Depression,” Journal of Nervous and Mental Disease 196, no. 2 (2008): 136–143, 10.1097/NMD.0b013e318162aa3f. 18277222

[34] N. J. Wiles , S. Hollinghurst , V. Mason , et al., “A Randomized Controlled Trial of Cognitive Behavioural Therapy as an Adjunct to Pharmacotherapy in Primary Care Based Patients With Treatment Resistant Depression: A Pilot Study,” Behavioural and Cognitive Psychotherapy 36, no. 1 (2008): 21–33, 10.1017/S135246580700389X.

[35] A. Nakagawa , D. Mitsuda , M. Sado , et al., “Effectiveness of Supplementary Cognitive‐Behavioral Therapy for Pharmacotherapy‐Resistant Depression: A Randomized Controlled Trial,” Journal of Clinical Psychiatry 78, no. 8 (2017): 1126–1135, 10.4088/JCP.15m10511. 28252882

[36] L. H. Souza , G. A. Salum , B. P. Mosqueiro , M. A. Caldieraro , T. A. Guerra , and M. P. Fleck , “Interpersonal Psychotherapy as Add‐On for Treatment‐Resistant Depression: A Pragmatic Randomized Controlled Trial,” Journal of Affective Disorders 193 (2016): 373–380, 10.1016/j.jad.2016.01.004. 26799332

[37] N. Wiles , L. Thomas , A. Abel , et al., “Cognitive Behavioural Therapy as an Adjunct to Pharmacotherapy for Primary Care Based Patients With Treatment Resistant Depression: Results of the CoBalT Randomised Controlled Trial,” Lancet 381, no. 9864 (2013): 375–384, 10.1016/S0140-6736(12)61552-9. 23219570

[38] N. J. Wiles , L. Thomas , N. Turner , et al., “Long‐Term Effectiveness and Cost‐Effectiveness of Cognitive Behavioural Therapy as an Adjunct to Pharmacotherapy for Treatment‐Resistant Depression in Primary Care: Follow‐Up of the CoBalT Randomised Controlled Trial,” Lancet Psychiatry 3, no. 2 (2016): 137–144, 10.1016/S2215-0366(15)00495-2. 26777773

[39] J. M. Town , A. Abbass , C. Stride , and D. Bernier , “A Randomised Controlled Trial of Intensive Short‐Term Dynamic Psychotherapy for Treatment Resistant Depression: The Halifax Depression Study,” Journal of Affective Disorders 214 (2017): 15–25, 10.1016/j.jad.2017.02.035. 28266318

[40] A. J. Rush , M. Fava , S. R. Wisniewski , et al., “Sequenced Treatment Alternatives to Relieve Depression (STAR*D): Rationale and Design,” Controlled Clinical Trials 25, no. 1 (2004): 119–142, 10.1016/s0197-2456(03)00112-0. 15061154

[41] J. E. Young , J. S. Klosko , and M. E. Weishaar , Schema Therapy: A Practitioner's Guide (Guilford Press, 2003), 449.

[42] Overview Depression in Adults: Treatment and Management Guidance NICE. NICE; 2022, https://www.nice.org.uk/guidance/ng222. 35977056

[43] D. Taylor , T. Barnes , and A. Young , The Maudsley Prescribing Guidelines in Psychiatry [Internet] (John Wiley & Sons, Ltd, 2025). 15th edn, https://onlinelibrary.wiley.com/doi/abs/10.1002/9781119870203.mpg016.

[44] 111.wales.nhs.uk [Internet] ., 2018 SSRIs (Selective Serotonin Reuptake Inhibitors), http://111.wales.nhs.uk:82/encyclopaedia/s/article/ssris(selectiveserotoninreuptakeinhibitors)/.

[45] T. Moreira , “Entangled Evidence: Knowledge Making in Systematic Reviews in Healthcare,” Sociology of Health and Illness 29, no. 2 (2007): 180–197, 10.1111/j.1467-9566.2007.00531.x. 17381812

[46] T. Greenhalgh , S. Ratnayake , R. Helm , et al., “The Mask Efficacy Controversy Reconsidered: A Systematic Review Using Mechanism‐Informed Narrative Synthesis,” 17 July 2026, Preprint (Version 1) available at Research Square, 10.21203/rs.3.rs-10307762/v1.

[47] S. Dosenovic , A. Dujmic , D. Nujic , et al., “Reasons and Factors Associated With Inconclusiveness of Systematic Reviews about Interventions for Neuropathic Pain,” Journal of Comparative Effectiveness Research 10, no. 1 (2021): 67–75, 10.2217/cer-2020-0165. 33355481

[48] J. D. Harris , M. P. Cote , A. Dhawan , E. Hohmann , and J. C. Brand , “Nearly One‐Third of Published Systematic Reviews and Meta‐Analyses Yield Inconclusive Conclusions: A Systematic Review,” Arthroscopy 37, no. 9 (2021): 2991–2998, 10.1016/j.arthro.2021.03.073. 33887412

[49] J. P. A. Ioannidis , “The Mass Production of Redundant, Misleading, and Conflicted Systematic Reviews and Meta‐Analyses,” Milbank Quarterly 94, no. 3 (2016): 485–514, 10.1111/1468-0009.12210. PMC5020151 27620683

